# Asymptomatic Hyperuricemia and the Kidney: Lessons from the URRAH Study

**DOI:** 10.3390/metabo15010011

**Published:** 2025-01-02

**Authors:** Cecilia Barnini, Elisa Russo, Giovanna Leoncini, Maria Carla Ghinatti, Lucia Macciò, Michela Piaggio, Francesca Viazzi, Roberto Pontremoli

**Affiliations:** 1 Department of Internal Medicine IV, Nephrology and Hypertension, Medical University Innsbruck, 6020 Innsbruck, Tirol, Austria; cecilia.barnini@gmail.com; 2 Department of Internal Medicine and Medical Specialties (DiMI), University of Genoa, 16132 Genoa, Italy; elisa.russo@unige.it (E.R.); giovanna.leoncini@unige.it (G.L.); lucia.maccio@edu.unige.it (L.M.); francesca.viazzi@unige.it (F.V.); 3 Unit of Nephrology, Dialysis and Transplantation, IRCCS Ospedale Policlinico San Martino, 16132 Genoa, Italy; michipiaggio98@gmail.com; 4 Internal Medicine Unit, IRCCS Ospedale Policlinico San Martino, 16132 Genoa, Italy; mariacarla.ghinatti@hsanmartino.it

**Keywords:** uric acid, kidney disease, cut-offs, cardiovascular risk

## Abstract

Chronic kidney disease (CKD) is a prevalent global health concern affecting approximately 850 million people worldwide, with a significant and rising mortality rate. CKD often coexists with hyperuricemia (HSUA), which is also increasingly common due to its association with hypertension, obesity, and diabetes. The interplay between hyperuricemia and CKD is complex; while in vitro studies and animal models support a role for uric acid mediating glomerular and tubule-interstitial damage, and HSUA has been shown to predict the onset and progression of CKD, the expectations of renal protection by the use of urate lowering treatment (ULT) are inconsistent. A significant challenge in managing asymptomatic HSUA in CKD patients lies in determining the appropriate SUA threshold values. Recent research, including the URRAH project, has sought to identify SUA cut-offs predictive of cardiovascular mortality, but these thresholds may vary depending on the severity of CKD. This variability complicates the establishment of universal guidelines for treating asymptomatic HSUA, leading to a lack of specific recommendations in clinical practice. In conclusion, while hyperuricemia is recognized as a prognostic factor for CKD and cardiovascular risk, more research is needed to refine the threshold values for SUA and to identify which patients may benefit from ULT. Stratification based on glomerular filtration rate may be necessary to tailor the treatments and improve outcomes in this population.

## 1. Chronic Kidney Disease: Epidemiology, Clinical Implications, and New Treatments

Chronic kidney disease (CKD) represents a growing global health issue. It is an extremely common disease with 850 million people worldwide estimated to be affected [1,2]. Consequently, the mortality related to this condition is also steadily increasing, and by 2040 CKD is expected to be the fifth leading cause of death [3]. This trend is likely due to the rising prevalence and incidence of some conditions like diabetes, hypertension, obesity, and elder age; in fact, the primary etiology of CKD differs globally, but, especially in high-income countries, diabetes and hypertension are the most common [4].

The range of CKD severity is wide, from mild to advanced forms, eventually leading to end-stage kidney disease (ESKD). CKD and ESKD are also associated with significant comorbidity and reduced quality of life [5], and CKD itself is a well-known, independent risk factor for cardiovascular (CV) events, hospitalization, and death from any cause [6]. Moreover, kidney diseases, and especially ESKD, are associated with elevated costs of care, with considerable consequences for health systems [7].

For these reasons, many efforts have been invested in the last decades in reducing CKD progression through the implementation of new medications able to slow the renal function decline. Significant benefits have been demonstrated with sodium-glucose cotransporter 2 (SGLT-2) inhibitors in both diabetic and non-diabetic patients [8,9,10] and with nonsteroidal selective mineralocorticoid receptor antagonist [11] and glucagon-like peptide 1 receptor agonist (GLP1RA) [12] in patients with diabetes.

Nevertheless, much remains to be done to limit the progression of renal diseases, and it still crucial to identify the risk factors for CKD in order to minimize their impact on kidney function decline through prevention and treatment. This concept has been clearly stated in the most recent Kidney Disease: Improving Global Outcomes (KDIGO) guideline for CKD, advocating for an early diagnosis of CKD, especially in high-risk subgroups such as those with hypertension, diabetes, and CV disease and for the implementation of new and existing treatments while optimizing the resources of health systems [13].

## 2. Hyperuricemia: Definition, Epidemiology, and Clinical Implications

Uric acid (UA) is the final product of purine breakdown; an imbalance in this metabolic process in terms of increased intake, overproduction, or reduced excretion results in hyperuricemia (HU). There is still an open debate about HU’s definition, and a universally accepted cut-off point does not exist.

Since the relationship between elevated UA levels and gout has been known for over a century [14], UA levels are traditionally measured with the aim of diagnosing gout, i.e., by serum uric acid (SUA) levels above 6.8 mg/dL [15]. This cut-off refers to the physicochemical definition of hyperuricemia, which expresses the saturation limit of monosodium urate, meaning the point when circulating uric acid precipitates as urate crystals [16]. As a matter of fact, an etiological role of high serum uric acid (HSUA) levels in developing diseases has been proven for conditions related to urate monosodium crystal deposition: gout [17], urate nephropathy [18,19], and nephrolithiasis [20]. These conditions are considered clinical manifestations of symptomatic hyperuricemia, and the chemical levels are widely accepted as a clinically relevant definition of HU.

Nevertheless, this definition has certain limitations and is not clearly sufficient to predict the development of diseases or guide treatment choices. In fact, most of the subjects with documented HSUA levels remain asymptomatic. Referring to gouty arthritis, for instance, serum urate concentration at baseline is a strong non-linear predictor of gout incidence, but only about half of those with very high levels (≥10 mg/dL) develop clinically evident gout over 15 years [21]. Therefore, the current clinical guidelines for the management of gout recommend treatment with urate-lowering therapy (ULT) with a strong level of evidence only in patients with clinical or radiological findings of crystal deposition or frequent gout flares [15,22]. Furthermore, referring to the treatment itself, the traditional upper threshold to diagnose HSUA concentration is not consistent with the therapeutic target of <6 mg/dL recommended by the guidelines for gout [15,22]; this is why it has been proposed to consider 6 mg/dL as the limit where HU starts [23]. This open debate on HU definitions makes it difficult to compare the results from various studies, affecting the homogeneity of consensus for the diagnosis and treatment of HU.

Moreover, the above-mentioned observations trigger the concept that HSUA levels are a necessary but insufficient factor to explain the clinical manifestation of urate deposits, thus involving genetics, environment, and individual inflammatory response. Regarding the environmental factors, for example, it is well known that some dietary habits [24,25], alcohol consumption [26], and obesity [27] contribute to the risk of gout, as does older age [28]. Additionally, the development of HU itself depends on purines intake and genetic predisposition, but it is also associated with insulin resistance [29] and visceral adiposity [30]. Consequently, evidence of both isolated hyperuricemia and symptomatic forms of HU are still significant, with an increasing tendency in the last few decades. HU is a very common biochemical abnormality with an estimated prevalence of 20% in high-income countries [31], probably due to increasing obesity, diabetes, and rising life expectancy.

Moving from these findings, new studies are investigating how SUAs interact with the CV and renal systems, proposing HU as an easily available biomarker and a potentially treatable risk factor for cardio-nephro-metabolic diseases. For this reason, it is necessary to find appropriate cut-offs to define HU, as it appears that conventional thresholds are unable to consider the full picture of the potential detrimental effects of HU.

## 3. Asymptomatic Hyperuricemia: Need to Revise the Concept?

Since patients with both symptomatic and asymptomatic forms of HU are frequently affected by cardio-nephro-metabolic comorbidities like hypertension, diabetes, metabolic syndrome, and kidney disease [32], it is crucial to understand whether these conditions often coexist because they share the same pathophysiological substrate or whether uric acid itself causes direct damage.

It has been demonstrated that the crystallized form of uric acid works as a danger signal of immune responses [33]; thus, it was speculated that the triggered inflammatory response could also explain the associated comorbidities [34]. Building on this concept, has been hypothesized that even under the already mentioned saturation level, circulating uric acid could exert a harmful effect on other organs, not directly mediated by crystal precipitation. In this setting, normally defined as asymptomatic hyperuricemia, it was proposed that elevated urate concentration could directly impact the CV system as well as kidney function [35]. HU is a frequent finding in people with hypertension [36], and it was also associated with an increased risk of incident hypertension [37]; a relationship was demonstrated also for metabolic syndrome [38], coronary artery disease [39], kidney disease onset [40], and CKD progression [41]. Interestingly, the association with hypertension was described even below the traditional cut-off for HU, showing a linear relation where the risk increases with increasing levels rather than starting after a certain threshold [37]. Similarly, a relationship with CV events has been observed for UA levels above 5.2 mg/dL [42], which means below the saturation point; thus, it is likely not dependent on monosodium crystals deposition. From the pathophysiological perspective, urate in its soluble form has a pro-oxidative effect [43] and is able to activate different pro-inflammatory pathways [44]. In this vein, it is possible to consider asymptomatic HU as a silent activator of the innate immune system, potentially implicated in certain systemic diseases’ mechanisms [45].

These factors explain why it was suggested to consider a cut-off of 6 mg/dL as a clinically relevant definition of HU able to better integrate the potential harmful consequences of HSUA levels [46].

However, it remains controversial whether uric acid is actively involved in the development of disease when it is not in the crystalline form, or whether it is simply a bystander that may act as a risk marker but not as a direct target for treatment or as a modifier of disease progression [47].

On the same note, the role of asymptomatic HU in CKD onset and progression is still debated [48]. In view of this uncertainty, the current guidelines for the management of CKD suggest that ULT should not be used in patients with CKD and asymptomatic hyperuricemia, with the aim of delaying CKD progression [13].

## 4. Uric Acid and the Kidney: From Physiological Regulation to Clinical Consequences

CKD and HSUA levels often occur together, although this finding does not necessarily infer a causality relationship. The kidney plays a central role in urate regulation, and serum urate concentrations depend on kidney function; indeed, urate is filtered in the glomerulus and then reabsorbed, secreted, and minimally excreted by the proximal tubule. Therefore, SUA levels are correlated with the glomerular filtration rate (GFR), and in the setting of CKD, with either glomerular filtration or tubular dysfunction, a rise in serum urate is expected; thus, HSUA is a frequent finding in people with CKD [49].

This correlation was initially described in individuals affected by gout, showing how rising rates of gout are correlated with decreasing renal function [50]; specifically, the occurrence of gout increases with the GFR decline, with a reported prevalence of over 30% in subjects with CKD stage 4 [51]. The reciprocal relation is also true, since the presence of gout is a risk a factor for incident CKD stage ≥ 3 [52].

Even though the coexistence of gout and CKD is recognized, it is still controversial to demonstrate the real impact of HSUA on kidney disease. Gout and CKD share some common risk factors like hypertension, diabetes, and elevated body mass index; moreover, some medications used for gout management could impact the kidney, like non-steroidal anti-inflammatory drugs (NSAIDs) [53], and, the other way around, treatments used in CKD patients, such as diuretics, may increase SUA concentration [54].

The attempt to understand the relationship between UA and kidney disease becomes even more complicated when referring to asymptomatic hyperuricemia. From the epidemiological perspective, the coexistence of CKD and HSUA has been depicted, as serum urate levels increase linearly with the decrease in glomerular filtration, with a threshold below 60 mL/min/1.73 m^2^ of estimated GFR (eGFR), independent of any other factor [55]. That was clearly described by the URRAH project, which evaluated the data of 26,971 individuals from the multicenter, retrospective, observational cohort of the URRAH study [56], showing that subjects with eGFR < 60 mL/min were ten times more likely to have HSUA compared with those with eGFR > 90 mL/min [57]. However, these observations could be just proof of the role of the kidney in urate metabolism, without any causative implication.

The first evidence of HU’s impact on the kidneys came from studies on renal involvement in patients affected by gout, where urate crystal deposits were identified in the renal medulla [58], leading to the definition of a specific disease identity named gouty nephropathy. In addition to that, though, subjects with gout presented less typical kidney lesions such as glomerulosclerosis, interstitial fibrosis, and arteriosclerosis [59]. Based on these observations, it was hypothesized that crystalline deposition was not the sole explanation for all findings. However, the challenging question is whether HSUA concentration affects the kidneys in a direct or indirect way, like by causing hypertension, which eventually leads to renal disease [60].

**Figure 1:**
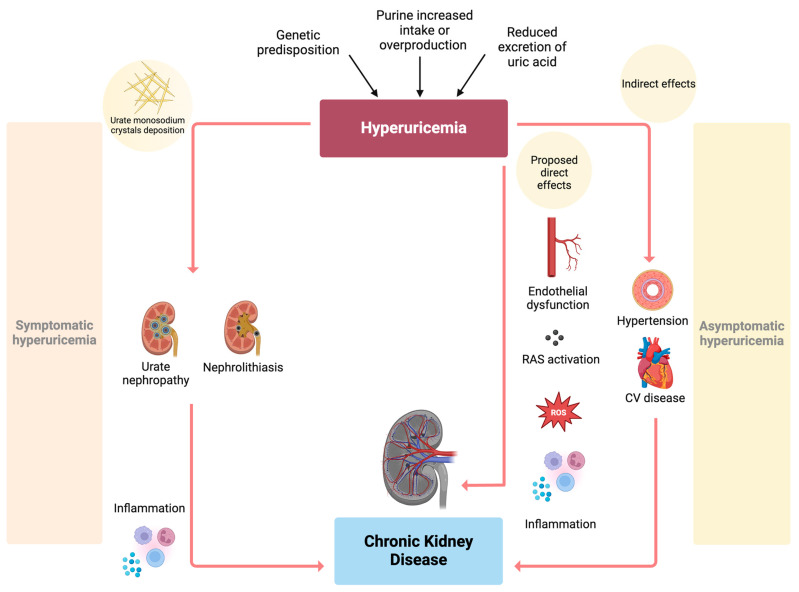
Kidney involvement in hyperuricemia. In the context of symptomatic hyperuricemia (**left side** of the figure), the etiological role of HSUA levels in developing kidney diseases is related to urate monosodium crystals deposition, which can lead to urate nephropathy and nephrolithiasis. In this setting, the crystallized form of uric acid works as an activator of immune responses, triggering inflammation and ultimately causing chronic kidney disease. It has been proposed that the asymptomatic form of hyperuricemia has relevant clinical implications for the kidneys as well (**right side** of the figure). One hypothesis is that HSUA levels may directly affect the kidneys through the pro-oxidative and pro-inflammatory effects of soluble urate, which activates the innate immune system. Moreover, UA can induce endothelial dysfunction and increase the activity of the renin-angiotensin system; these proposed mechanisms might eventually lead to arteriosclerosis, glomerulosclerosis, and interstitial fibrosis. In addition to that, UA may have an indirect effect on renal function, i.e., by inducing hypertension and CV disease, which subsequently cause CKD. Abbreviations. RAS: renin-angiotensin system; ROS: reactive oxygen species; HSUA: high serum uric acid; UA: uric acid; CV: cardiovascular; CKD: chronic kidney disease.

As a matter of fact, evidence from animal models demonstrates that HSUA levels correlate with endothelial dysfunction and increased activity of the renin-angiotensin system, resulting in arteriolosclerosis and glomerular hypertension [61,62]; through analogous mechanisms, elevated uric acid even seemed able to accelerate CKD progression [63] (Figure 1).

## 5. Hyperuricemia and CKD Progression: The Chicken–Egg Problem

Although a co-occurrence between CKD and HSUA is well documented, it is challenging to understand how the two variables interplay, and the separate contributions of both in terms of the pathogenic mechanism is still not completely clear. Moving from pathophysiological mechanisms, several hypotheses have been proposed to explain the potential role of uric acid in kidney damage [64]. Evidence similar to that found in experimental models has been detected in humans: in summary, urate might cause renal dysfunction because of the induction of a fibrogenic cascade elicited by inflammation [65], which leads to interstitial fibrosis. On the other hand, uric acid can mediate endothelial dysfunction through the activation of the renin-angiotensin system (RAS) [66] and by inducing the proliferation and modification of vascular smooth cells [67], eventually leading to glomerulosclerosis.

As a matter of fact, asymptomatic HSUA has been shown to be a risk factor for both the onset and progression of CKD. Strong positive associations between serum urate levels and the risk of CKD have been consistently shown in observational studies [40,68,69]. Epidemiological research indicates that HSUA independently predicts the development of CKD in individuals with normal kidney function, both in the general population and in diabetic patients [70,71]. High SUA levels have been shown to more than double the risk of developing a new estimated glomerular filtration rate (eGFR) < 60 mL/min/1.73 m^2^ in 13,964 Type 2 diabetes (T2D) patients, and they are known to be associated with the presence and severity of albuminuria [71]. In individuals with normal renal function, elevated UA levels appeared as an independent predictor of kidney disease and microalbuminuria development [72].

Moreover, SUA was demonstrated as a significant independent predictor of developing CKD in kidney donors [73], supporting previous studies suggesting that HSUA may play a pathogenic role in renal disease.

Conversely, results from Mendelian randomization studies testing genetic variants known to increase UA levels have not found an increased risk of CKD in this population, and therefore, do not support the role of HU as a causative factor in CKD [74,75].

The finding that HSUA precedes and predicts CKD development challenges the idea that its negative impact in CKD patients is solely due to reduced kidney function leading to impaired excretion and retention. Interestingly, a differential nephrotoxic and CV impact of SUA based on kidney function was found in several wide cohorts [76]. This might partly explain the inconsistent findings regarding the role of urate-lowering therapy (ULT) in renal progression.

While high SUA levels are proposed as a risk factor leading to CKD, this association is more apparent in those with normal kidney function and less so in patients with existing kidney disease in terms of CKD progression [77,78]. In a 7-year follow-up study involving 177 patients with non-diabetic CKD, no evidence of uric acid as an independent predictor for CKD progression was detected [79]. Similarly, some other studies have not found uric acid to be an independent risk factor for CKD progression, particularly in advanced stages of the disease or in kidney transplant patients [80,81]. This suggests that once the kidney disease is chronic and the damage is irreversible, the progression of CKD does not depend on UA levels.

The relationship between SUA and kidney function may also be influenced by gender, with women being more susceptible to uric acid-induced kidney function decline. Some studies have shown that HSUA predicts end-stage renal disease (ESKD) primarily in women [82].

Lastly, both very low and very high uric acid levels have been associated with poor kidney outcomes, possibly due to low levels being indicative of malnutrition, which is linked to worse CKD outcomes [83,84]. This complexity may contribute to the varying results seen in studies on uric acid and kidney disease progression.

## 6. Treating Hyperuricemia and Renal Protection: Where Do We Stand?

Today, the possibility that pharmacologically induced lowering of SUA may improve kidney outcomes remains open due to methodological weaknesses in the available trials that make the results inconclusive. Moreover, the threshold values of SUA that should be applied to define asymptomatic HSUA and therefore be treated, especially in the presence of CKD, is an open issue.

Goicoechea et al. [85] were the first to show in a randomized controlled trial (RCT) that allopurinol could independently slow the progression of renal disease in CKD patients over an average of 2 years compared with controls. In a later post hoc intention-to-treat analysis, the authors confirmed that allopurinol’s positive impact on kidney function and CV risk persisted over a median follow-up of 7 years [86].

In a recent RCT involving 467 patients with stage 3 CKD over 2 years, the FEATHER study, febuxostat did not slow CKD progression compared with a placebo in the whole cohort. Notably, the drug seemed to be effective in patients with milder kidney impairment (those without proteinuria or with lower serum creatinine levels) [87].

The largest randomized study available (FREED Study), with 1070 hyperuricemic patients, compared ULT with non-ULT for renal protection, showing that febuxostat effectively lowered SUA levels, which was linked to a reduction in the primary composite cerebrovascular, cardiovascular, and renal endpoints [88].

Two recently published RCTs were unable to prove the nephroprotective role of allopurinol, but this lack of effect has several possible explanations. The CKD-FIX trial [89] randomized 369 CKD stage 3 and 4 patients with albuminuria or eGFR decline rate ≥ 3.0 mL/min/1.73 m^2^ in the preceding 12 months. The change in eGFR did not differ significantly between the allopurinol group and the placebo group (−3.33 mL/min/1.73 m^2^ vs. −3.23 mL/min/1.73 m^2^, respectively) [89]. The high-risk population, the heterogeneous surrogate outcome, and the insufficient power due to an incomplete enrollment and the high percentage of discontinuation of the trial regimen (25–30%), could explain the inconsistency of the results. The other recent trial that failed to demonstrate a beneficial effect of allopurinol on CKD progression was the PERL study, in which 267 patients with long-term type 1 diabetes were treated with allopurinol/placebo for 3 years [90]. The mean decrease in the measured GFR was −3.0 mL/min/1.73 m^2^/year with allopurinol and −2.5 mL/min/1.73 m^2^/year with placebo, meaning no evidence of clinically meaningful benefits of SUA reduction with allopurinol on kidney outcomes among a population with a very long duration of diabetes (mean 34.6 years). These two cohorts depict a figure of very high risk of progression, a setting in which clinicians cannot expect the desired effect from ULT.

Conversely, the most recent metanalysis on this topic shows that ULT plays an important role in delaying the progression of renal impairment in CKD patients with asymptomatic HSUA, with no significant racial differences and in both early-stage CKD patients (eGFR  ≥ 45 mL/min/1.73 m^2^) and late-stage CKD patients (eGFR  <  45 mL/min/1.73 m^2^) [91]. Thus, it appears that many of the studies completed so far have insufficient sample sizes, short follow-up times, or design limitations (such as patient heterogeneity, high discontinuation rates of trial drugs, etc.), leading to challenges to the robustness of the conclusions. Maybe further RCT studies with different inclusion criteria and longer follow-up periods will provide more reliable evidence confirming whether ULT has renal protective effects in CKD patients with asymptomatic HSUA.

Another source of confusion on the topic lies in cut-off values for treating asymptomatic HSUA. The strong connection between uric acid and CV risk [92], wherein metabolic dysregulation and heart remodeling (two factors closely related to kidney disease) play a key role, complicates the issue. With the aim of answering this question, the Uric acid Right for heArt Health (URRAH) project was designed by the working group on uric acid and CV risk of the Italian Society of Hypertension. Operational cut-offs for SUA (i.e., 5.6 and 4.7 mg/dL) were identified in terms of CV and all-cause mortality in a high-risk general cohort [93]. Nevertheless, it is still unclear which threshold values should be applied to define asymptomatic HSUA in the presence of CKD, given the relationship between eGFR and SUA [94], while also considering the independent pathogenetic contribution of each one of these variables to CV risk [80,95].

The search for the cut-off to define asymptomatic HSUA in various settings has been the focus of much research in recent years [96,97,98]. A recent URRAH sub-analysis demonstrated that patients with CKD stage 3A (eGFR lower than 60 mL/min), show a six-fold greater incidence of fatal CV events over a median follow-up time of 10 years compared with those with preserved renal function. Moreover, recent studies show that SUA becomes a significant correlate of unfavorable outcomes in patients with reduced eGFR only at serum concentrations greater than what is observed in subjects with normal renal function [50]. In fact, eGFR-specific predictive SUA levels for CV mortality have been identified: ROC analysis indicated that SUA values of 4.8 and 6.8 mg/dL, respectively, could be a valuable threshold to predict future mortality in patients with low eGFR values, namely, between 60 and 90 and below 60 mL/min [99]. In summary, HSUA is demonstrated to be an additional potential risk factor for CV mortality in CKD patients [82,100] but with different cut-off values. More longitudinal studies are needed to confirm and extend these results.

Within this context, an intriguing hypothesis, first put forward by Borghi et al. [101,102,103], maintains that the detrimental effect of HSUA may be accounted for, beyond its severity, by the prevalent specific mechanisms by which UA accumulates within the body, i.e., by means of increased XO activity or, as in the case of CKD patients, mainly by accumulation due to inadequate kidney clearance. As intriguing as this hypothesis may be, it is difficult to discern pathophysiological mechanisms when dealing with hyperuricemic patients in clinical practice.

Considering all the above and the considerable remaining areas of clinical uncertainty, the current absence of specific recommendations for SUA treatment in CKD patients is understandable.

## 7. New Treatments for HSUA and CKD: Considering the Cardio-Nephro-Metabolic Continuum

SGLT-2 inhibitors (SGLT-2i) represent a foundational treatment for diabetes, heart failure (HF), and CKD. Given the above-mentioned implications of HU in cardio-nephro-metabolic conditions, the possible effect of SGLTs on uric acid levels was investigated in order to understand whether they might exert additional benefits [104,105].

In patients with T2DM, two post hoc analyses from the CANVAS program and the EMPA-REG OUTCOME trial [106] showed that canagliflozin and empagliflozin, respectively, reduce UA levels and gout episodes or prescriptions of ULTs. Similarly, subjects with HF included in two large RCTs and treated with dapagliflozin had lower rates of initiation of antigout agents and ULTs [107].

To add further evidence in the CKD population, a meta-analysis of eight RCTs with SGLT-2i showed that these agents were able to significantly reduce SUA levels compared with placebo, but when subgroup analyses based on eGFR were performed, SGLT-2i reduced uric acid concentrations only in participants with CKD stages 1 and 2 [108]. A recent exploratory analysis from the EMPA-KIDNEY trial showed how empagliflozin treatment resulted in a significant reduction in SUA levels, but the effect was larger in non-diabetic participants and, accordingly with the previous meta-analyses, for higher eGFR levels [109]. Overall, empagliflozin did not reduce the occurrence of gout. Furthermore, empagliflozin’s benefits on reducing CKD progression were not dependent on UA concentrations.

Even though the net advantage of SGLT-2i in treating HU in CKD populations needs further studies, it is interesting to understand the way these agents can reduce UA levels. The main mechanism seems to be an acceleration in uric acid excretion [110]; specifically, UA is normally reabsorbed in the proximal tubule by the URAT1 (uric acid transporter 1) [111], but it is also transported to the blood via GLUT9 [112]. Since SGLT-2i inhibits GLUT9, leading to glycosuria, the urate absorption is also compromised, hence increasing UA excretion [113,114]. For this reason, analogously to the glycosuric effect, the urate excretion induced by SGLT-2i inhibitors depends on renal function: this may explain the reported reduced effect in lowering UA levels in CKD patients with lower eGFR. Although the potential benefits of new drugs such as SGLT2 inhibitors on uric acid and kidney health are significant, current data are limited, and the effects seem to be influenced by kidney function status. Thus, further investigations could provide additional insights, translating preliminary evidence into clinical applications.

## 8. Conclusions

Hyperuricemia is undoubtedly an important prognostic factor for diabetes and metabolism dysregulation, hypertension, kidney disease onset and progression, and CV risk. A reduction in GFR is associated with higher SUA levels, and in this population, the cut-off values appear to be different. Some studies have shown a benefit of ULT on both CV risk and the progression of kidney disease, particularly in patients who have not yet developed severe and/or longstanding damage. According to recent findings, the cut-off values for defining asymptomatic hyperuricemia are lower than those traditionally used for CV and overall mortality risk. However, these cut-off values may not be applicable to all populations, and stratification based on GFR may be useful. Today, it remains to be determined which patients will derive renal and CV benefits from ULT and which cut-off values should be used. Nevertheless, the possibility to delay the decline of kidney function in patients with CKD through ULT deserves attention.

## Data Availability

Data is available with in the article.

## References

[1] Kovesdy C.P. (2022). Epidemiology of chronic kidney disease: An update 2022. Kidney Int. Suppl..

[2] Jager K.J., Kovesdy C., Langham R., Rosenberg M., Jha V., Zoccali C. (2019). A single number for advocacy and communication—Worldwide more than 850 million individuals have kidney diseases. Kidney Int..

[3] Foreman K.J., Marquez N., Dolgert A., Fukutaki K., Fullman N., McGaughey M., Pletcher M.A., Smith A.E., Tang K., Yuan C.-W. (2018). Forecasting life expectancy, years of life lost, and all-cause and cause-specific mortality for 250 causes of death: Reference and alternative scenarios for 2016-40 for 195 countries and territories. Lancet Lond. Engl..

[4] Couser W.G., Remuzzi G., Mendis S., Tonelli M. (2011). The contribution of chronic kidney disease to the global burden of major noncommunicable diseases. Kidney Int..

[5] Webster A.C., Nagler E.V., Morton R.L., Masson P. (2017). Chronic Kidney Disease. Lancet.

[6] Go A.S., Chertow G.M., Fan D., McCulloch C.E., Hsu C. (2004). Chronic kidney disease and the risks of death, cardiovascular events, and hospitalization. N. Engl. J. Med..

[7] Luyckx V.A., Al-Aly Z., Bello A.K., Bellorin-Font E., Carlini R.G., Fabian J., Garcia-Garcia G., Iyengar A., Sekkarie M., van Biesen W. (2021). Sustainable Development Goals relevant to kidney health: An update on progress. Nat. Rev. Nephrol..

[8] Perkovic V., Jardine M.J., Neal B., Bompoint S., Heerspink H.J.L., Charytan D.M., Edwards R., Agarwal R., Bakris G., Bull S. (2019). Canagliflozin and Renal Outcomes in Type 2 Diabetes and Nephropathy. N. Engl. J. Med..

[9] Heerspink H.J.L., Stefánsson B.V., Correa-Rotter R., Chertow G.M., Greene T., Hou F.-F., Mann J.F.E., McMurray J.J.V., Lindberg M., Rossing P. (2020). Dapagliflozin in Patients with Chronic Kidney Disease. N. Engl. J. Med..

[10] (2023). The EMPA-KIDNEY Collaborative Group Empagliflozin in Patients with Chronic Kidney Disease. N. Engl. J. Med..

[11] Bakris G.L., Agarwal R., Anker S.D., Pitt B., Ruilope L.M., Rossing P., Kolkhof P., Nowack C., Schloemer P., Joseph A. (2020). Effect of Finerenone on Chronic Kidney Disease Outcomes in Type 2 Diabetes. N. Engl. J. Med..

[12] Perkovic V., Tuttle K.R., Rossing P., Mahaffey K.W., Mann J.F.E., Bakris G., Baeres F.M.M., Idorn T., Bosch-Traberg H., Lausvig N.L. (2024). Effects of Semaglutide on Chronic Kidney Disease in Patients with Type 2 Diabetes. N. Engl. J. Med..

[13] Stevens P.E., Ahmed S.B., Carrero J.J., Foster B., Francis A., Hall R.K., Herrington W.G., Hill G., Inker L.A., Kazancıoğlu R. (2024). KDIGO 2024 Clinical Practice Guideline for the Evaluation and Management of Chronic Kidney Disease. Kidney Int..

[14] Garrod A.B. (1848). Observations on certain pathological conditions of the blood and urine, in gout, rheumatism, and Bright’s disease. Med.-Chir. Trans..

[15] FitzGerald J.D., Dalbeth N., Mikuls T., Brignardello-Petersen R., Guyatt G., Abeles A.M., Gelber A.C., Harrold L.R., Khanna D., King C. (2020). 2020 American College of Rheumatology Guideline for the Management of Gout. Arthritis Care Res..

[16] Bardin T., Richette P. (2014). Definition of hyperuricemia and gouty conditions. Curr. Opin. Rheumatol..

[17] Narang R.K., Dalbeth N. (2020). Pathophysiology of Gout. Semin. Nephrol..

[18] Shimada M., Johnson R.J., May W.S., Lingegowda V., Sood P., Nakagawa T., Van Q.C., Dass B., Ejaz A.A. (2009). A novel role for uric acid in acute kidney injury associated with tumour lysis syndrome. Nephrol. Dial. Transplant..

[19] Lusco M.A., Fogo A.B., Najafian B., Alpers C.E. (2017). AJKD Atlas of Renal Pathology: Gouty Nephropathy. Am. J. Kidney Dis..

[20] Moe O.W., Abate N., Sakhaee K. (2002). Pathophysiology of uric acid nephrolithiasis. Endocrinol. Metab. Clin. N. Am..

[21] Dalbeth N., Phipps-Green A., Frampton C., Neogi T., Taylor W.J., Merriman T.R. (2018). Relationship between serum urate concentration and clinically evident incident gout: An individual participant data analysis. Ann. Rheum. Dis..

[22] Richette P., Doherty M., Pascual E., Barskova V., Becce F., Castañeda-Sanabria J., Coyfish M., Guillo S., Jansen T.L., Janssens H. (2017). 2016 updated EULAR evidence-based recommendations for the management of gout. Ann. Rheum. Dis..

[23] Bardin T. (2015). Hyperuricemia starts at 360 micromoles (6 mg/dL). Jt. Bone Spine.

[24] Zhang Y., Chen C., Choi H., Chaisson C., Hunter D., Niu J., Neogi T. (2012). Purine-rich foods intake and recurrent gout attacks. Ann. Rheum. Dis..

[25] Sacks F.M., Svetkey L.P., Vollmer W.M., Appel L.J., Bray G.A., Harsha D., Obarzanek E., Conlin P.R., Miller E.R., Simons-Morton D.G. (2001). Effects on blood pressure of reduced dietary sodium and the Dietary Approaches to Stop Hypertension (DASH) diet. DASH-Sodium Collaborative Research Group. N. Engl. J. Med..

[26] Zhang Y., Woods R., Chaisson C.E., Neogi T., Niu J., McAlindon T.E., Hunter D. (2006). Alcohol Consumption as a Trigger of Recurrent Gout Attacks. Am. J. Med..

[27] McAdams DeMarco M.A., Maynard J.W., Huizinga M.M., Baer A.N., Köttgen A., Gelber A.C., Coresh J. (2011). Obesity and younger age at gout onset in a community-based cohort. Arthritis Care Res..

[28] Mikuls T.R., Farrar J.T., Bilker W.B., Fernandes S., Schumacher H.R., Saag K.G. (2005). Gout epidemiology: Results from the UK General Practice Research Database, 1990–1999. Ann. Rheum. Dis..

[29] Facchini F., Chen Y.D., Hollenbeck C.B., Reaven G.M. (1991). Relationship between resistance to insulin-mediated glucose uptake, urinary uric acid clearance, and plasma uric acid concentration. JAMA.

[30] Takahashi S., Yamamoto T., Tsutsumi Z., Moriwaki Y., Yamakita J., Higashino K. (1997). Close correlation between visceral fat accumulation and uric acid metabolism in healthy men. Metabolism.

[31] Zhu Y., Pandya B.J., Choi H.K. (2011). Prevalence of gout and hyperuricemia in the US general population: The National Health and Nutrition Examination Survey 2007–2008. Arthritis Rheum..

[32] Pillinger M.H., Goldfarb D.S., Keenan R.T. (2010). Gout and its comorbidities. Bull. NYU Hosp. Jt. Dis..

[33] Shi Y., Evans J.E., Rock K.L. (2003). Molecular identification of a danger signal that alerts the immune system to dying cells. Nature.

[34] Rock K.L., Kataoka H., Lai J.-J. (2013). Uric acid as a danger signal in gout and its comorbidities. Nat. Rev. Rheumatol..

[35] Feig D.I., Kang D.-H., Johnson R.J. (2008). Uric Acid and Cardiovascular Risk. N. Engl. J. Med..

[36] Cannon P.J., Stason W.B., Demartini F.E., Sommers S.C., Laragh J.H. (1966). Hyperuricemia in primary and renal hypertension. N. Engl. J. Med..

[37] Grayson P.C., Kim S.Y., LaValley M., Choi H.K. (2011). Hyperuricemia and incident hypertension: A systematic review and meta-analysis. Arthritis Care Res..

[38] King C., Lanaspa M.A., Jensen T., Tolan D.R., Sánchez-Lozada L.G., Johnson R.J. (2018). Uric Acid as a Cause of the Metabolic Syndrome. Contrib. Nephrol..

[39] Tuttle K.R., Short R.A., Johnson R.J. (2001). Sex differences in uric acid and risk factors for coronary artery disease. Am. J. Cardiol..

[40] Li L., Yang C., Zhao Y., Zeng X., Liu F., Fu P. (2014). Is hyperuricemia an independent risk factor for new-onset chronic kidney disease?: A systematic review and meta-analysis based on observational cohort studies. BMC Nephrol..

[41] Chonchol M., Shlipak M.G., Katz R., Sarnak M.J., Newman A.B., Siscovick D.S., Kestenbaum B., Carney J.K., Fried L.F. (2007). Relationship of uric acid with progression of kidney disease. Am. J. Kidney Dis..

[42] Niskanen L.K., Laaksonen D.E., Nyyssönen K., Alfthan G., Lakka H.-M., Lakka T.A., Salonen J.T. (2004). Uric acid level as a risk factor for cardiovascular and all-cause mortality in middle-aged men: A prospective cohort study. Arch. Intern. Med..

[43] Zhou Y., You H., Zhang A., Jiang X., Pu Z., Xu G., Zhao M. (2020). Lipoxin A4 attenuates uric acid-activated, NADPH oxidase-dependent oxidative stress by interfering with translocation of p47phox in human umbilical vein endothelial cells. Exp. Ther. Med..

[44] Braga T.T., Forni M.F., Correa-Costa M., Ramos R.N., Barbuto J.A., Branco P., Castoldi A., Hiyane M.I., Davanso M.R., Latz E. (2017). Soluble Uric Acid Activates the NLRP3 Inflammasome. Sci. Rep..

[45] Joosten L.A.B., Crişan T.O., Bjornstad P., Johnson R.J. (2020). Asymptomatic hyperuricaemia: A silent activator of the innate immune system. Nat. Rev. Rheumatol..

[46] Desideri G., Castaldo G., Lombardi A., Mussap M., Testa A., Pontremoli R., Punzi L., Borghi C. (2014). Is it time to revise the normal range of serum uric acid levels?. Eur. Rev. Med. Pharmacol. Sci..

[47] Johnson R.J., Bakris G.L., Borghi C., Chonchol M.B., Feldman D., Lanaspa M.A., Merriman T.R., Moe O.W., Mount D.B., Sanchez Lozada L.G. (2018). Hyperuricemia, Acute and Chronic Kidney Disease, Hypertension, and Cardiovascular Disease: Report of a Scientific Workshop Organized by the National Kidney Foundation. Am. J. Kidney Dis..

[48] Du L., Zong Y., Li H., Wang Q., Xie L., Yang B., Pang Y., Zhang C., Zhong Z., Gao J. (2024). Hyperuricemia and its related diseases: Mechanisms and advances in therapy. Signal Transduct. Target. Ther..

[49] Johnson R.J., Sanchez Lozada L.G., Lanaspa M.A., Piani F., Borghi C. (2023). Uric Acid and Chronic Kidney Disease: Still More to Do. Kidney Int. Rep..

[50] Krishnan E. (2012). Reduced Glomerular Function and Prevalence of Gout: NHANES 2009-10. PLoS ONE.

[51] Juraschek S.P., Kovell L.C., Miller E.R., Gelber A.C. (2013). Association of kidney disease with prevalent gout in the United States in 1988–1994 and 2007–2010. Semin. Arthritis Rheum..

[52] Roughley M., Sultan A.A., Clarson L., Muller S., Whittle R., Belcher J., Mallen C.D., Roddy E. (2018). Risk of chronic kidney disease in patients with gout and the impact of urate lowering therapy: A population-based cohort study. Arthritis Res. Ther..

[53] Baker M., Perazella M.A. (2020). NSAIDs in CKD: Are They Safe?. Am. J. Kidney Dis..

[54] Ben Salem C., Slim R., Fathallah N., Hmouda H. (2017). Drug-induced hyperuricaemia and gout. Rheumatology.

[55] Cirillo M., Laurenzi M., Mancini M., Zanchetti A., Lombardi C., De Santo N.G. (2006). Low glomerular filtration in the population: Prevalence, associated disorders, and awareness. Kidney Int..

[56] Desideri G., Virdis A., Casiglia E., Borghi C., Working Group on Uric Acid and Cardiovascular Risk of the Italian Society of Hypertension (2018). Exploration into Uric and Cardiovascular Disease: Uric Acid Right for heArt Health (URRAH) Project, A Study Protocol for a Retrospective Observational Study. High Blood Press. Cardiovasc. Prev..

[57] Russo E., Viazzi F., Pontremoli R., Barbagallo C.M., Bombelli M., Casiglia E., Cicero A.F.G., Cirillo M., Cirillo P., Desideri G. (2022). Association of uric acid with kidney function and albuminuria: The Uric Acid Right for heArt Health (URRAH) Project. J. Nephrol..

[58] Bardin T., Nguyen Q.D., Tran K.M., Le N.H., Do M.D., Richette P., Letavernier E., Correas J.-M., Resche-Rigon M. (2021). A cross-sectional study of 502 patients found a diffuse hyperechoic kidney medulla pattern in patients with severe gout. Kidney Int..

[59] Talbott J.H., Terplan K.L. (1960). The kidney in gout. Medicine.

[60] Yü T.-F., Berger L. (1982). Impaired renal function in gout. Am. J. Med..

[61] Nakagawa T., Mazzali M., Kang D.-H., Kanellis J., Watanabe S., Sanchez-Lozada L.G., Rodriguez-Iturbe B., Herrera-Acosta J., Johnson R.J. (2003). Hyperuricemia Causes Glomerular Hypertrophy in the Rat. Am. J. Nephrol..

[62] Sánchez-Lozada L.G., Soto V., Tapia E., Avila-Casado C., Sautin Y.Y., Nakagawa T., Franco M., Rodríguez-Iturbe B., Johnson R.J. (2008). Role of oxidative stress in the renal abnormalities induced by experimental hyperuricemia. Am. J. Physiol.-Ren. Physiol..

[63] Kang D.-H., Nakagawa T., Feng L., Watanabe S., Han L., Mazzali M., Truong L., Harris R., Johnson R.J. (2002). A Role for Uric Acid in the Progression of Renal Disease. J. Am. Soc. Nephrol..

[64] Russo E., Verzola D., Cappadona F., Leoncini G., Garibotto G., Pontremoli R., Viazzi F. (2021). The role of uric acid in renal damage—A history of inflammatory pathways and vascular remodeling. Vessel Plus.

[65] Braga T.T., Foresto-Neto O., Camara N.O.S. (2020). The role of uric acid in inflammasome-mediated kidney injury. Curr. Opin. Nephrol. Hypertens..

[66] Perlstein T.S., Gumieniak O., Hopkins P.N., Murphey L.J., Brown N.J., Williams G.H., Hollenberg N.K., Fisher N.D.L. (2004). Uric acid and the state of the intrarenal renin-angiotensin system in humans. Kidney Int..

[67] Zhen H., Gui F. (2017). The role of hyperuricemia on vascular endothelium dysfunction. Biomed. Rep..

[68] Kumagai T., Ota T., Tamura Y., Chang W.X., Shibata S., Uchida S. (2017). Time to target uric acid to retard CKD progression. Clin. Exp. Nephrol..

[69] Weiner D.E., Tighiouart H., Elsayed E.F., Griffith J.L., Salem D.N., Levey A.S. (2008). Uric acid and incident kidney disease in the community. J. Am. Soc. Nephrol. JASN.

[70] Obermayr R.P., Temml C., Gutjahr G., Knechtelsdorfer M., Oberbauer R., Klauser-Braun R. (2008). Elevated Uric Acid Increases the Risk for Kidney Disease. J. Am. Soc. Nephrol..

[71] De Cosmo S., Viazzi F., Pacilli A., Giorda C., Ceriello A., Gentile S., Russo G., Rossi M.C., Nicolucci A., Guida P. (2015). Serum Uric Acid and Risk of CKD in Type 2 Diabetes. Clin. J. Am. Soc. Nephrol..

[72] Lee J.E., Kim Y.-G., Choi Y.-H., Huh W., Kim D.J., Oh H.Y. (2006). Serum uric acid is associated with microalbuminuria in prehypertension. Hypertension.

[73] Bravo R.C., Gamo M.B., Lee H.H., Yoon Y.E., Han W.K. (2017). Investigating Serum Uric Acid as a Risk Factor in the Development of Delayed Renal Recovery in Living Kidney Donors. Transplant. Proc..

[74] Jordan D.M., Choi H.K., Verbanck M., Topless R., Won H.-H., Nadkarni G., Merriman T.R., Do R. (2019). No causal effects of serum urate levels on the risk of chronic kidney disease: A Mendelian randomization study. PLoS Med..

[75] Li X., Meng X., Timofeeva M., Tzoulaki I., Tsilidis K.K., Ioannidis P.A., Campbell H., Theodoratou E. (2017). Serum uric acid levels and multiple health outcomes: Umbrella review of evidence from observational studies, randomised controlled trials, and Mendelian randomisation studies. BMJ.

[76] Toyama T., Furuichi K., Shimizu M., Hara A., Iwata Y., Sakai N., Perkovic V., Kobayashi M., Mano T., Kaneko S. (2015). Relationship between Serum Uric Acid Levels and Chronic Kidney Disease in a Japanese Cohort with Normal or Mildly Reduced Kidney Function. PLoS ONE.

[77] Tsai C.-W., Lin S.-Y., Kuo C.-C., Huang C.-C. (2017). Serum Uric Acid and Progression of Kidney Disease: A Longitudinal Analysis and Mini-Review. PLoS ONE.

[78] Uchida S., Chang W.X., Ota T., Tamura Y., Shiraishi T., Kumagai T., Shibata S., Fujigaki Y., Hosoyamada M., Kaneko K. (2015). Targeting Uric Acid and the Inhibition of Progression to End-Stage Renal Disease--A Propensity Score Analysis. PLoS ONE.

[79] Sturm G., Kollerits B., Neyer U., Ritz E., Kronenberg F. (2008). Uric acid as a risk factor for progression of non-diabetic chronic kidney disease? The Mild to Moderate Kidney Disease (MMKD) Study. Exp. Gerontol..

[80] Liu W.-C., Hung C.-C., Chen S.-C., Yeh S.-M., Lin M.-Y., Chiu Y.-W., Kuo M.-C., Chang J.-M., Hwang S.-J., Chen H.-C. (2012). Association of Hyperuricemia with Renal Outcomes, Cardiovascular Disease, and Mortality. Clin. J. Am. Soc. Nephrol..

[81] Kalil R.S., Carpenter M.A., Ivanova A., Gravens-Mueller L., John A.A., Weir M.R., Pesavento T., Bostom A.G., Pfeffer M.A., Hunsicker L.G. (2017). Impact of Hyperuricemia on Long-term Outcomes of Kidney Transplantation: Analysis of the FAVORIT Study. Am. J. Kidney Dis..

[82] Srivastava A., Kaze A.D., McMullan C.J., Isakova T., Waikar S.S. (2018). Uric Acid and the Risks of Kidney Failure and Death in Individuals with CKD. Am. J. Kidney Dis..

[83] Chang W., Uchida S., Qi P., Zhang W., Wang X., Liu Y., Han Y., Li J., Xu H., Hao J. (2020). Decline in serum uric acid predicts higher risk for mortality in peritoneal dialysis patients—A propensity score analysis. J. Nephrol..

[84] Kanda E., Muneyuki T., Kanno Y., Suwa K., Nakajima K. (2015). Uric Acid Level Has a U-Shaped Association with Loss of Kidney Function in Healthy People: A Prospective Cohort Study. PLoS ONE.

[85] Goicoechea M., De Vinuesa S.G., Verdalles U., Ruiz-Caro C., Ampuero J., Rincón A., Arroyo D., Luño J. (2010). Effect of Allopurinol in Chronic Kidney Disease Progression and Cardiovascular Risk. Clin. J. Am. Soc. Nephrol..

[86] Goicoechea M., Garcia De Vinuesa S., Verdalles U., Verde E., Macias N., Santos A., Pérez De Jose A., Cedeño S., Linares T., Luño J. (2015). Allopurinol and Progression of CKD and Cardiovascular Events: Long-term Follow-up of a Randomized Clinical Trial. Am. J. Kidney Dis..

[87] Kimura K., Hosoya T., Uchida S., Inaba M., Makino H., Maruyama S., Ito S., Yamamoto T., Tomino Y., Ohno I. (2018). Febuxostat Therapy for Patients with Stage 3 CKD and Asymptomatic Hyperuricemia: A Randomized Trial. Am. J. Kidney Dis..

[88] Kojima S., Matsui K., Hiramitsu S., Hisatome I., Waki M., Uchiyama K., Yokota N., Tokutake E., Wakasa Y., Jinnouchi H. (2019). Febuxostat for Cerebral and CaRdiorenovascular Events PrEvEntion StuDy. Eur. Heart J..

[89] Badve S.V., Pascoe E.M., Tiku A., Boudville N., Brown F.G., Cass A., Clarke P., Dalbeth N., Day R.O., De Zoysa J.R. (2020). Effects of Allopurinol on the Progression of Chronic Kidney Disease. N. Engl. J. Med..

[90] Doria A., Galecki A.T., Spino C., Pop-Busui R., Cherney D.Z., Lingvay I., Parsa A., Rossing P., Sigal R.J., Afkarian M. (2020). Serum Urate Lowering with Allopurinol and Kidney Function in Type 1 Diabetes. N. Engl. J. Med..

[91] Luo Y., Song Q., Li J., Fu S., Yu W., Shao X., Li J., Huang Y., Chen J., Tang Y. (2024). Effects of uric acid-lowering therapy (ULT) on renal outcomes in CKD patients with asymptomatic hyperuricemia: A systematic review and meta-analysis. BMC Nephrol..

[92] Maloberti A., Mengozzi A., Russo E., Cicero A.F.G., Angeli F., Agabiti Rosei E., Barbagallo C.M., Bernardino B., Bombelli M., Cappelli F. (2023). The Results of the URRAH (Uric Acid Right for Heart Health) Project: A Focus on Hyperuricemia in Relation to Cardiovascular and Kidney Disease and its Role in Metabolic Dysregulation. High Blood Press. Cardiovasc. Prev..

[93] Virdis A., Masi S., Casiglia E., Tikhonoff V., Cicero A.F.G., Ungar A., Rivasi G., Salvetti M., Barbagallo C.M., Bombelli M. (2020). Identification of the Uric Acid Thresholds Predicting an Increased Total and Cardiovascular Mortality Over 20 Years. Hypertension.

[94] Oliveira I.O., Mintem G.C., Oliveira P.D., Freitas D.F., Brum C.B., Wehrmeister F.C., Gigante D.P., Horta B.L., Menezes A.M.B. (2020). Uric acid is independent and inversely associated to glomerular filtration rate in young adult Brazilian individuals. Nutr. Metab. Cardiovasc. Dis..

[95] Neri L., Rocca Rey L.A., Lentine K.L., Hinyard L.J., Pinsky B., Xiao H., Dukes J., Schnitzler M.A. (2011). Joint Association of Hyperuricemia and Reduced GFR on Cardiovascular Morbidity: A Historical Cohort Study Based on Laboratory and Claims Data from a National Insurance Provider. Am. J. Kidney Dis..

[96] Masulli M., D’Elia L., Angeli F., Barbagallo C.M., Bilancio G., Bombelli M., Bruno B., Casiglia E., Cianci R., Cicero A.F.G. (2022). Serum uric acid levels threshold for mortality in diabetic individuals: The URic acid Right for heArt Health (URRAH) project. Nutr. Metab. Cardiovasc. Dis..

[97] Perticone M., Maio R., Shehaj E., Gigliotti S., Caroleo B., Suraci E., Sciacqua A., Andreozzi F., Perticone F. (2023). Sex-related differences for uric acid in the prediction of cardiovascular events in essential hypertension. A population prospective study. Cardiovasc. Diabetol..

[98] Ma C., Yu H., Zhang W., Fu H., Wan G., Yang G., Zhang X., Xie R., Lv Y., Zhang J. (2023). High-normal serum uric acid predicts macrovascular events in patients with type 2 diabetes mellitus without hyperuricemia based on a 10-year cohort. Nutr. Metab. Cardiovasc. Dis..

[99] Russo E., Viazzi F., Pontremoli R., Barbagallo C.M., Bombelli M., Casiglia E., Cicero A.F.G., Cirillo M., Cirillo P., Desideri G. (2021). Serum Uric Acid and Kidney Disease Measures Independently Predict Cardiovascular and Total Mortality: The Uric Acid Right for Heart Health (URRAH) Project. Front. Cardiovasc. Med..

[100] Madero M., Sarnak M.J., Wang X., Greene T., Beck G.J., Kusek J.W., Collins A.J., Levey A.S., Menon V. (2009). Uric Acid and Long-term Outcomes in CKD. Am. J. Kidney Dis..

[101] Borghi C., Piani F. (2020). Uric acid and estimate of renal function. Let’s stick together. Int. J. Cardiol..

[102] Agnoletti D., Cicero A.F.G., Borghi C. (2021). The Impact of Uric Acid and Hyperuricemia on Cardiovascular and Renal Systems. Cardiol. Clin..

[103] Borghi C., Agabiti-Rosei E., Johnson R.J., Kielstein J.T., Lurbe E., Mancia G., Redon J., Stack A.G., Tsioufis K.P. (2020). Hyperuricaemia and gout in cardiovascular, metabolic and kidney disease. Eur. J. Intern. Med..

[104] Caruso I., Giorgino F. (2022). SGLT-2 inhibitors as cardio-renal protective agents. Metabolism.

[105] Lamprea-Montealegre J.A., Shlipak M.G., Estrella M.M. (2021). Chronic kidney disease detection, staging and treatment in cardiovascular disease prevention. Heart.

[106] Ferreira J.P., Inzucchi S.E., Mattheus M., Meinicke T., Steubl D., Wanner C., Zinman B. (2022). Empagliflozin and uric acid metabolism in diabetes: A post hoc analysis of the EMPA-REG OUTCOME trial. Diabetes Obes. Metab..

[107] Butt J.H., Docherty K.F., Claggett B.L., Desai A.S., Petersson M., Langkilde A.M., de Boer R.A., Hernandez A.F., Inzucchi S.E., Kosiborod M.N. (2023). Association of Dapagliflozin Use with Clinical Outcomes and the Introduction of Uric Acid–Lowering Therapy and Colchicine in Patients with Heart Failure with and Without Gout: A Patient-Level Pooled Meta-analysis of DAPA-HF and DELIVER. JAMA Cardiol..

[108] Zhang L., Zhang F., Bai Y., Huang L., Zhong Y., Zhang X. (2024). Effects of sodium-glucose cotransporter-2 (SGLT-2) inhibitors on serum uric acid levels in patients with chronic kidney disease: A systematic review and network meta-analysis. BMJ Open Diabetes Res. Care.

[109] Mayne K.J., Sardell R.J., Staplin N., Judge P.K., Zhu D., Sammons E., Cherney D.Z.I., Green J.B., Levin A., Pontremoli R. (2024). Empagliflozin lowers serum uric acid in chronic kidney disease: Exploratory analyses from the EMPA-KIDNEY trial. Nephrol. Dial. Transplant..

[110] Chino Y., Samukawa Y., Sakai S., Nakai Y., Yamaguchi J., Nakanishi T., Tamai I. (2014). SGLT2 inhibitor lowers serum uric acid through alteration of uric acid transport activity in renal tubule by increased glycosuria. Biopharm. Drug Dispos..

[111] Enomoto A., Kimura H., Chairoungdua A., Shigeta Y., Jutabha P., Ho Cha S., Hosoyamada M., Takeda M., Sekine T., Igarashi T. (2002). Molecular identification of a renal urate–anion exchanger that regulates blood urate levels. Nature.

[112] Li S., Sanna S., Maschio A., Busonero F., Usala G., Mulas A., Lai S., Dei M., Orrù M., Albai G. (2007). The GLUT9 Gene Is Associated with Serum Uric Acid Levels in Sardinia and Chianti Cohorts. PLoS Genet..

[113] Bailey C.J. (2019). Uric acid and the cardio-renal effects of SGLT2 inhibitors. Diabetes Obes. Metab..

[114] Leoncini G., Russo E., Bussalino E., Barnini C., Viazzi F., Pontremoli R. (2021). SGLT2is and Renal Protection: From Biological Mecha-nisms to Real-World Clinical Benefits. Int. J. Mol. Sci..

